# Draft Genome Sequence of VIM-2-Producing Multidrug-Resistant *Pseudomonas aeruginosa* ST175, an Epidemic High-Risk Clone

**DOI:** 10.1128/genomeA.00112-13

**Published:** 2013-04-11

**Authors:** Esther Viedma, Carlos Juan, Joaquín R. Otero, Antonio Oliver, Fernando Chaves

**Affiliations:** Servicio de Microbiología Clínica, Hospital Universitario 12 de Octubre, Madrid, Spaina;; Servicio de Microbiología and Unidad de Investigación, Hospital Universitario Son Espases, Palma de Mallorca, Spainb

## Abstract

The VIM-2-producing multidrug-resistant high-risk clone *Pseudomonas aeruginosa* sequence type (ST) 175 was isolated in the setting of a large outbreak in Hospital Universitario 12 de Octubre (Spain) from 2007 to 2010. This strain was resistant to all β-lactams, fluoroquinolones, and aminoglycosides, with the exception of amikacin, and has become an endemic clone in our institution.

## GENOME ANNOUNCEMENT

*Pseudomonas aeruginosa* is among the most relevant nosocomial pathogens, frequently causing severe infections in immunocompromised and critically ill patients, due to its ubiquitous nature, ability to colonize and survive in hospital reservoirs, and remarkable intrinsic antibiotic resistance and virulence (1). The versatility of *P. aeruginosa* to combine mutation-driven and horizontally acquired resistance mechanisms has led to the emergence of strains that are resistant to nearly all antimicrobials, dramatically compromising our therapeutic options to treat the infections caused by these pathogens (2, 3). Particularly concerning are the increasing reports of outbreaks, in multiple hospitals from several countries, of strains producing metallo-β-lactamases (MBL), with VIM-2 being the dominant MBL variant in Spain and worldwide (4–7). Moreover, most of these outbreaks are caused by a very limited number of *P. aeruginosa* genotypes, denominated international high-risk clones (8). Thus, deciphering the genetic determinants driving the success of these clones is crucial for the establishment of control and treatment strategies.

The *P. aeruginosa* strain PA21_ST175 was isolated from a blood culture in the setting of a large outbreak by a VIM-2-producing multidrug-resistant *P. aeruginosa* clone. The outbreak affected a total of 104 patients and persisted in our hospital for at least 34 months despite the control measures that were implemented. This strain was responsible for over half of the infections or colonizations by multidrug-resistant *P. aeruginosa* from 2007 to 2010, reaching 76% in the last year of the period studied (9). Moreover, it belonged to the international high-risk clone of sequence type (ST) 175 and was resistant to all beta-lactams, fluoroquinolones, and aminoglycosides, with the exception of amikacin. Particularly noteworthy, the strain produced a VIM-2 MBL and an aminoglycoside-modifying enzyme (AAC6′Ib) located in a class I integron (9). 

 Whole-genome shotgun sequencing was performed using a Roche 454 Junior sequencer. A total of 164,273,685 bp was obtained from Roche 454, providing approximately 22-fold coverage and 368,892 reads, with a G+C content of 66.1%.

Sequences obtained were used for *de novo* assembly using Newbler Assembler v2.7 (Roche). The draft genome sequence consists of 100 contigs with an *N*_50_ contig size of 278,105 nucleotides and a total length of 6,889,935 bp. Sequences were annotated using the NCBI Prokaryotic Genomes Automatic Annotation Pipeline (PGAAP) (http://www.ncbi.nlm.nih.gov/) and the Rapid Annotations using Subsystems Technology (RAST) server (10), yielding a total of 6,488 coding DNA sequence (CDS) genes and 58 tRNAs. This approach highlighted the presence of up to 152 genes related to antibiotic and antiseptic resistance, including the previously characterized *bla*_VIM-2_. Several chromosomal mutations involved in antibiotic resistance, including many of those previously reported for other ST175 lineages (11), were also detected.

Ongoing comparative genomic analysis with other widespread high-risk clones, such as ST111 or ST235 (12), and nonepidemic multidrug-resistant strains will help to elucidate the secret of the success of these international multidrug-resistant clonal lineages, a crucial step in the establishment of global control and treatment strategies to combat them.

### Nucleotide sequence accession number. 

The draft genome sequence of *P. aeruginosa* ST175 has been included in the GenBank Whole-Genome Shotgun (WGS) database under the accession no. AOIH00000000.
